# Addressing the Financial Consequences of Cancer: Qualitative Evaluation of a Welfare Rights Advice Service

**DOI:** 10.1371/journal.pone.0042979

**Published:** 2012-08-10

**Authors:** Suzanne Moffatt, Emma Noble, Martin White

**Affiliations:** Institute of Health and Society, Newcastle University, Newcastle upon Tyne, Tyne and Wear, United Kingdom; Academic Medical Center, Netherlands

## Abstract

**Background:**

The onset, treatment and trajectory of cancer is associated with financial stress among patients across a range of health and welfare systems and has been identified as a significant unmet need. Welfare rights advice can be delivered effectively in healthcare settings, has the potential to alleviate financial stress, but has not yet been evaluated. We present an evaluation of a welfare rights advice intervention designed to address the financial consequences of cancer.

**Methods:**

Descriptive study of welfare outcomes among 533 male and 641 female cancer patients and carers aged 4–95 (mean 62) years, who accessed the welfare rights advice service in North East England between April 2009 and March 2010; and qualitative interview study of a maximum variation sample of 35 patients and 9 carers.

**Results:**

Over two thirds of cancer patients and carers came from areas of high socio-economic deprivation. Welfare benefit claims were successful for 96% of claims made and resulted in a median increase in weekly income of £70.30 ($109.74, €84.44). Thirty-four different types of benefits or grants were awarded. Additional resources were perceived to lessen the impact of lost earnings, help offset costs associated with cancer, reduce stress and anxiety and increase ability to maintain independence and capacity to engage in daily activities, all of which were perceived to impact positively on well-being and quality of life. Key barriers to accessing benefit entitlements were knowledge, system complexity, eligibility concerns and assumptions that health professionals would alert patients to entitlements.

**Conclusions:**

The intervention proved feasible, effectively increased income for cancer patients and was highly valued. Addressing the financial sequelae of cancer can have positive social and psychological consequences that could significantly enhance effective clinical management and suitable services should be routinely available. Further research is needed to evaluate health outcomes definitely and assess cost-effectiveness.

## Introduction

Advances in cancer treatment have led to increases in long term survival for many types of cancer. With more people living with cancer, a greater focus on the psycho-social implications of cancer [1], [2] and assistance with financial matters have been identified as significant unmet needs [3]–[4]. The onset, treatment and trajectory of cancer is associated with financial stress among patients across a range of health care and welfare systems [5]. The financial difficulties associated with cancer may be due to temporary or permanent loss of earnings, as well as the additional costs associated with cancer, and depend on cancer type, occupation and wealth. This has been documented throughout different health and welfare systems [6]–[9]. Considerable degrees of financial stress were found in US studies of patients with terminal cancer; with even greater levels of financial hardship reported among African Americans, and among those with high care needs [5]. In 2005, half of all personal bankruptcies in the USA were related to medical expenses [10]. Despite a well-developed welfare state and National Health Service, it is estimated that in the UK nine out of ten cancer patients’ households experience loss of income as a direct result of cancer [11].

The incidence of many cancers varies by socioeconomic group in the UK [12]–[13] and other countries [14]–[16]. Across the developed world, there is a consistent pattern of higher mortality from cancer among lower socio-economic groups [17]. Those who are already financially disadvantaged therefore suffer a poorer outcome of cancer. Lower income is associated with worse health but it also reduces the capacity to cope with the consequences of ill-health [18]. Those in lower socio-economic groups with fewer financial resources, therefore, face a greater struggle to cope with the cancer trajectory which can involve any combination of debilitating treatments, recurrence over long periods, difficulties returning to work, and end of life care.

Many advanced welfare states have payment systems to counteract the loss of earnings or additional outgoings that result from ill-health. Although the UK benefits system is designed to provide financial assistance for people with health problems, the system is regarded as highly complex and difficult to access unaided for both patients and health professionals [19]. The system has special rules to accommodate rapidly progressing terminal cancers where life expectancy is six months or less. In practice, many patients with rapidly progressing fatal cancers do not receive timely advice and miss out on receiving their entitlements [20]. Current clinical guidance from the UK National Institute for Health and Clinical Excellence (NICE) Guidance on Cancer Services recommends that the topic of finance is raised with cancer patients, moreover that, ‘*patients and carers should be offered assistance to obtain benefits for which they are potentially eligible by professionals who are knowledgeable about the benefits system’* [21] (p88). In the UK, welfare rights advisors who may be employed by local government or charities are the profession with expertise on the state welfare system and who could therefore best provide expertise in assisting patients and carers. However the recent National Cancer Patient Experience Survey found that only 50% of patients who said it was necessary, received information about financial help [22].

Welfare rights advice, a non-statutory advocacy service funded by local government and/or charities, has been proposed as an intervention that can increase income from welfare payments for those with health problems [23]. Some welfare benefits are ‘means tested’ where eligibility depends on level of household income and assets, but others, particularly health-related benefits, are provided on the basis of health or care needs and not means tested. By directly increasing access to financial and other resources (such as aids and adaptations for the home), welfare rights advice enhances a patient’s ability to cope with the material consequences of illness and therefore has the potential to reduce socio-economic inequalities in quality of life following a cancer diagnosis [24]. In the UK, welfare rights advice services have also been shown to have a positive impact on local economies [25].

In the UK, welfare rights advice services are not routinely available for health professionals, such as General Practitioners or Cancer Nurse Specialists, to refer patients to, nor for patients to access independently. Yet, qualitative research with people over state pension age has demonstrated that obtaining additional resources can reduce stress, increase individuals’ ability to cope with health problems and improve quality of life [26]. A systematic review of 55 studies of the health, social and financial impacts of welfare rights advice delivered in healthcare settings demonstrated that welfare rights advice services increased the uptake of financial benefits [27]. There was, however, little evidence that welfare rights advice resulted in measurable health or social benefits due to lack of high quality studies. A meta-ethnography of social support for people affected by cancer concluded that the need for financial advice and its impact is under-researched [2].

While the difficulties associated with the financial consequences of cancer have received some attention [1]–[6], there is no research on the impact of initiatives designed to deliver financial advice and/or increase access to financial resources. In the UK, welfare rights advice services enable people with cancer to access hitherto unclaimed financial resources. This paper reports a qualitative evaluation of such a service, delivered in a health care setting for cancer patients, addressing the question: what impact do welfare rights advice services have on the quality of life and wellbeing of people with cancer?

## Methods

### Ethics Statement

The study was approved by Sunderland NHS Local Research Ethics Committee via the Integrated Research Application System. Research governance approval was obtained from the Research Management and Governance Unit of County Durham & Tees Valley Primary Care Trust. Written informed consent was obtained from all participants who took part in interviews.

### Study Setting, Intervention and Study Population

In June 2008 Durham County Council, in collaboration with a major UK cancer charity, Macmillan Cancer Support, appointed three experienced welfare rights advisors to provide a dedicated service for people with cancer and their carers across County Durham in North East England. County Durham has a population of 504,900 that includes urban, semi-rural and remote rural populations, as well as areas of significant socio-economic deprivation and poor health. Around one third of the population live in areas among the most deprived in England, with only ten per cent living in areas among the least deprived [28]. Life expectancy in County Durham for males and females and early deaths from cancer are significantly worse than the average for England [28]. In terms of cancer type, the highest incidence rates in men are for lung, prostate and colorectal cancers; for women the highest incidence rates are for breast, lung and colorectal cancers [29].

The welfare rights advice service was designed to be freely accessible, so that individuals could self refer, as well as be referred by health, social care or voluntary (charity) sector professionals. The staff work in a range of voluntary (charity) sector and National Health Service (NHS) locations throughout the county, including in-patient and out-patient hospital locations and primary care, as well as providing the service via home visits, supported by further contacts by email and telephone. The service comprises a full personal finance and welfare benefit eligibility assessment, followed by assistance to claim entitlements, follow-up work and representation at appeals and tribunals (for initially rejected claims). The welfare rights advisors also undertake outreach work to voluntary and community groups in order to facilitate awareness among the wider public. In addition, they carry out awareness training for health, social care and voluntary sector staff in order to increase referral rates and to enable these staff to deliver basic benefit information to optimise the reach of the service.

### Study Population and Benefit Outcome Data

The study population was all cancer patients who accessed the service between April 2009 and March 2010. Benefit eligibility and social and demographic data were collected from cancer patients using a standard questionnaire by welfare rights advisers at the initial consultation. To assign a measure of socio-economic position, indices of multiple deprivation were calculated at the lower super output area level [30] matched to individual household unit postcodes. Name, address, ethnicity, postcode were then removed to anonymise the data set before transfer to the research team. Individual deprivation scores were assigned to fifths of the distribution of the Index of Multiple Deprivation nationally for further analyses. Age was calculated from year of birth to a mid-year point and assigned to ten-year age bands. Data were checked, cleaned and analysed in SPSS version 17.0. [31]. The outcome variables were welfare benefits obtained (see appendix S1). Data were analysed descriptively to assess the benefit outcomes by age, sex and multiple deprivation indices, and to compare financial gains for men and women above and below the national pension age.

### Service User Involvement

At the outset of the study, we worked with various cancer patient groups approached through the Northern Cancer Network (now North of England Cancer Network), to include a ‘user engagement’ group in the study. Despite considerable effort, we found that individuals with cancer preferred to remain within their own cancer support group and did not wish to join a user group for the study. However, on completion of the study, we ensured that the findings were disseminated to all the groups and individuals who we were in contact with.

### Qualitative Data

In order to investigate the financial consequences of cancer and the impact of the welfare rights advice service from patient and carer perspectives, qualitative methods were used [32]. Semi-structured interviews, were undertaken with the broadest practicable range of participants using maximum variation sampling [33]. A topic guide was developed based on available literature concerning the financial consequences of cancer [1]–[6] and previous fieldwork with older people in poor health [26]. The guide covered, benefits-related issues, impact of cancer on work, family and finances, and, the impact of welfare rights advice on quality of life and wellbeing. The sampling frame was all cancer patients accessing the service who agreed to be approached for interview. Interviews were undertaken in participants’ homes, after informed consent was obtained and ranged in length from 22 to 100****minutes, were digitally recorded and transcribed in full. Criteria to achieve maximum variation sampling were: age, sex, cancer type and Index of Multiple Deprivation. Those who were seriously unwell were not invited for interview. Data collection continued until data saturation was reached [34].

Following close reading of transcripts, a conceptual framework was devised and data coded using the coding procedure in Nvivo Version 7 [35]. Using the Framework method, the data were charted systematically so that participants’ circumstances, experiences and views could be compared within and across groups in a framework derived from their own accounts [36]. Constant comparison [37]–[38] and deviant case analysis [39] were used to enhance internal validity [40].

## Results

### Study Population and Benefit Outcomes

The welfare rights advisers conducted 1231 consultations with 1174 individuals (57 individuals were seen more than once) between April 2009 and March 2010 within County Durham in North East England. Table 1 summarises the social and demographic characteristics of the sample. The median age of service users was 62 years. Sixty-nine per cent of the sample lived in areas within the highest and second highest fifths of the Index of Multiple Deprivation (i.e. the 40 per cent most deprived areas in England).

**Table 1:** Social and demographic characteristics of 1174 individuals accessing Macmillan welfare rights advice service (April 2009–March 2010).

*Variable*	*Category*	*Number*	*Percentage*
**Sex**	Male	533	45.4
n = 1174	Female	641	54.6
**Age Bands**	0–19	5	0.4
n = 1118 (95.2%)	20–29	10	0.9
Missing 56 (4.8%)	30–39	37	3.3
	40–49	144	12.9
	50–59	254	22.7
	60–69	299	25.7
	70–79	274	24.6
	80–89	88	7.9
	90–99	7	0.6
**Marital Status**	Single (Never married)	118	10.1
n = 1174	Married	721	61.4
	Co-habiting	58	4.9
	Divorced	61	5.2
	Separated	23	2.0
	Widowed	140	11.9
	Civil Partnership	1	0.1
	Unknown	52	4.4
**Fifths of the Indices of Multiple Deprivation (IMD)**	1 = 1–6496 (most deprived)	450	38.7
n = 1163 (99.1%)	2 = 6497–12993	358	30.8
Missing 11 (0.9%)	3 = 12994–19489	156	13.4
	4 = 19490–25986	122	10.5
	5 = 25987–32482 (least deprived)	77	6.6
**Employment Status**	Retired	551	46.9
n = 1174	Unemployed	349	29.7
	Employed	151	12.9
	Self Employed	20	1.7
	Dependent Child	2	0.2
	Unknown	101	8.6

Most participants were referred to the welfare rights service by a health professional in primary care (28%) or secondary care (17%), a considerable proportion referred themselves (23%), the Macmillan Cancer Information Service referred a further 11% and the remainder were referred by self help groups, social services, other welfare rights services family and friends, hospice staff and Citizens Advice Bureau (9%). It was not possible to establish the referral source for 12%.

For the period April 2009 - March 2010, 1540 benefit claims were made, of which 1475 (96%) were successful. UK state benefits are linked together in a system of conditional entitlements; thus some individuals received more than one state benefit. In total 34 different types of benefits were claimed. Table 2 shows the eight most frequently claimed benefits, and their relationship to age, sex and Indicator of Multiple Deprivation. Table 3 shows that substantial amounts of benefits were awarded to those above and below national pension age (median weekly awards of £70.30 ($109.74, €84.44) and £115.50 ($180.25, €138.82) respectively).

**Table 2:** Socio demographic characteristics of patients and carers making the most frequent resolved financial claims (April 2009–March 2010).

Number (%) by type of benefit																	
		DLA* (care) (n = 277)		DLA* (mobility) (n = 203)		Attendance Allowance(n = 191)		Macmillan Grants (n = 229)		Pension Credit (GuaranteeCredit) (n = 89)		Council TaxBenefit (n = 69)		ESA**ContributionBased (n = 62)		Carers Allowance	
Gender	*Male*	144	(52.0)	103	(50.7)	94	(49.2)	95	(41.5)	43	(48.3)	26	(37.7)	33	(53.2)	25	(46.3)
	*Female*	130	(46.9)	99	(48.8)	93	(48.7)	129	(56.3)	43	(48.3)	41	(59.4)	29	(46.8)	29	(53.7)
	*Unknown*	3	(1.1)	1	(0.5)	4	(2.1)	5	(2.2)	3	(3.4)	2	(2.9)	0	(0)	0	(0)
	*Total*	277	(100)	203	(100)	191	(100)	229	(100)	89	(100)	69	(100)	62	(100)	54	(100)
Age	*Range*	11–87		11–64		65–91		11–84		60–87		39–83		38–64		11–87	
	*Mean*	56.2		53.8		74.5		56.6		70.3		62		52.6		55.9	
	*Median*	59		57		74		57		70		61		54		57	
	*Total*	266		193		184		228		85		67		60		48	
	*Missing*	11	(4.0)	10	(4.9)	7	(3.7)	1	(0.4)	4	(4.5)	2	(2.9)	2	(3.2)	6	(11.1)
IMD (fifths)	*1 (most deprived)*	109	(39.6)	82	(40.6)	59	(31.1)	101	(44.3)	39	(43.9)	31	(44.9)	19	(30.6)	24	(46.2)
	*2*	82	(29.9)	59	(29.2)	65	(34.2)	83	(36.4)	31	(34.8)	22	(31.9)	25	(40.3)	15	(28.8)
	*3*	37	(13.5)	26	(12.9)	27	(14.2)	24	(10.5)	13	(14.6)	11	(15.9)	7	(11.3)	8	(15.4)
	*4*	29	(10.5)	23	(11.4)	24	(12.6)	18	(7.9)	4	(4.5)	4	(5.9)	7	(11.3)	4	(7.7)
	*5 (least deprived)*	18	(6.5)	12	(5.9)	15	(7.9)	2	(0.9)	2	(2.2)	1	(1.4)	4	(6.5)	1	(1.9)
	*Total*	275	(100)	202	(100)	190	(100)	228	(100)	89	(100)	69	(100)	62	(100)	52	(100)
	*Missing (n)*	2	(0.7)	1	(0.5)	1	(0.5)	1	(0.5)	1	(0.4)	0	(0)	0	(0)	2	(3.7)

* DLA  =  Disability Living Allowance.

** ESA  =  Employment Support Allowance (see appendix S1).

**Table 3:** Median amount of weekly benefit awarded per person (£,$,€) (April 2009–March 2010).*

Median (range) amount of weekly benefits awarded per person (£,$,€)				
			Above state pension age	Below state pension age
			(n = 279)	(n = 292)
Men	Median (Range)	GBP	70.30 (3.00–235.00)	119.40 (3.00–417.50)
(n = 280)		US Dollar	109.74 (4.68–364.38)	185.13 (4.68–651.72)
		Euro	84.44 (3.63–284.55)	144.57 (363–501.71)
Women	Median (Range)	GBP	70.30 (6.00–406.20)	101.20 (2.00–292.20)
(n = 291)		US Dollar	109.74 (9.30–629.39)	156.92 (3.12–452.76)
		Euro	84.44 (7.26–491.81)	122.53 (2.40–354.16)
All claimants	Median (Range)	GBP	70.30 (3.00–406.20)	115.50 (2.00–417.40)
(n = 571)		US Dollar	109.74 (4.68–633.93)	180.25 (3.12–651.41)
		Euro	84.44 (3.63–488.14)	138.82 (2.40–501.60)

* All amounts calculated at highest rate paid and length of entitlement may have varied over 12 month period.

### Qualitative Findings

Two hundred and fifty-nine people consented to take part in interviews, from which 35 cancer patients and nine carers were purposively sampled and interviewed; 27 were interviewed alone; eight with a carer and one carer alone. Characteristics of the 35 cancer patients and one carer are summarised in Table 4. Patients interviewed were aged from 30 to over 80 years, and mostly resided in areas within the most deprived two-fifths of areas and had a range of cancer types. Most patients of working age were off work due to cancer or another illness.

**Table 4:** Demographic factors and cancer type of interview sample.*

Category of information collected		No. of participants
Male		19
Female		17
Age	*30–39*	5
	*40–49*	9
	*50–59*	8
	*60–69*	6
	*70–79*	7
	*80+*	1
Fifths of IMD	*1 (most deprived)*	15
	*2*	12
	*3*	5
	*4*	3
	*5 (least deprived)*	1
Rural/Urban Indicator	*3 (Village-sparse)*	1
	*5 (Urban-less sparse)*	20
	*6 (Town & Fringe-less sparse)*	10
	*7 (Village-less sparse)*	5
Employment Status	*Back to work*	3
	*In work part time over 16 hours*	1
	*In work part time under 16 hours*	1
	*Year off work to care*	1
	*Off work long term (cancer)*	7
	*Off work short term (cancer)*	6
	*Off work long term other morbidity*	5
	*Redundant*	2
	*Retired (over state pension age)*	8
	*Retired (under state pension age)*	1
	*Unknown*	1
Cancer type [International Classificationof Diseases (ICD-10) section]		
C00–C14	*Malignant neoplasms of lip, oral cavity and pharynx*	2
C15–C26	*Malignant neoplasms of digestive organs*	7
C30–C39	*Malignant neoplasms of respiratory and intrathoracic organs*	5
C40–C41	*Malignant neoplasms of bone and articular cartilage*	2
C43–C44	*Melanoma and other malignant neoplasms of skin*	1
C50–C50	*Malignant neoplasms of breast*	4
C51–C58	*Malignant neoplasms of female genital organs*	1
C60–C63	*Malignant neoplasms of male genital organs*	4
C73–C75	*Malignant neoplasms of thyroid and other endocrine glands*	2
C81–C96	*Malignant neoplasms, stated or presumed to be primary, of lymphoid, haematopoietic* *and related tissue*	8
C97–C97	*Malignant neoplasms of independent (primary) multiple sites*	1

* Demographic information collected for 35 interviewees and one carer.

#### Impact of receiving welfare rights advice

Receiving welfare rights advice had three immediate consequences. Firstly, lessening the impact of lost earnings as a result of temporary or permanent cessation of work, by assisting people to apply for and receive illness and incapacity benefits.

… she [wife] said she was down to about £300 … my account was down to about £200 … how are we going to pay the bills for about seven or eight months of our lives … [having cancer] is just financial devastation really … without the help coming through [welfare rights advice] this place [house] would have been on the market, it would have gone you know. (P155, male, aged 45–49 years).

Secondly, receiving additional resources offset the additional costs associated with cancer, which included travel and parking, dietary requirements, heating and clothing costs, as well as adaptations to the home and paying for extra help.

It has [helped] in some respects because there are things that I’ve got to pay out for … especially now because I’ve got to rely on other people … I can’t drive for two years, right, so [a friend] takes me to the hospital. Well, my Disability Living Allowance gives me the freedom to say, right, there’s £10, you know, thanks for taking us sort of thing. And I don’t feel obligated … you know, it’s £10 to her. (P052, female, aged 40–49).

[following additional resources] … we can afford the salmon. He’s getting good fish into him … before [the additional money], you know, it used to be fish cakes or fish fingers, something like that. Couple of fish fingers, couple of chips, and that was your dinner. Now he gets a piece of salmon and a dinner, four or five veg and potatoes and things like that you see. Which makes it a lot better for both of us, we’re both healthier eating. (C012, female, carer).

Thirdly, the welfare rights advisors facilitated access to a comprehensive range of on-going advice, information, practical support and onward referral to a wide variety of other agencies including support organisations, money and debt advice and charities. This was particularly important throughout a period of significant change in health and financial circumstances.

… he’s never ever slept for all the year … since having the cancer. And he’s been depressed about it. But he’s never ever gone to the doctor with depression … But when the welfare rights advisor came out that’s when he was saying about not sleeping and just sitting about all the time … and she said ‘do you know, go back and see your doctor and explain everything to him’. So the doctor sent him to a counsellor. So he’s been a couple of times to see a counsellor as well, which, you know, it was the welfare rights adviser that said go. (C010, female, carer).

**Figure 1:**
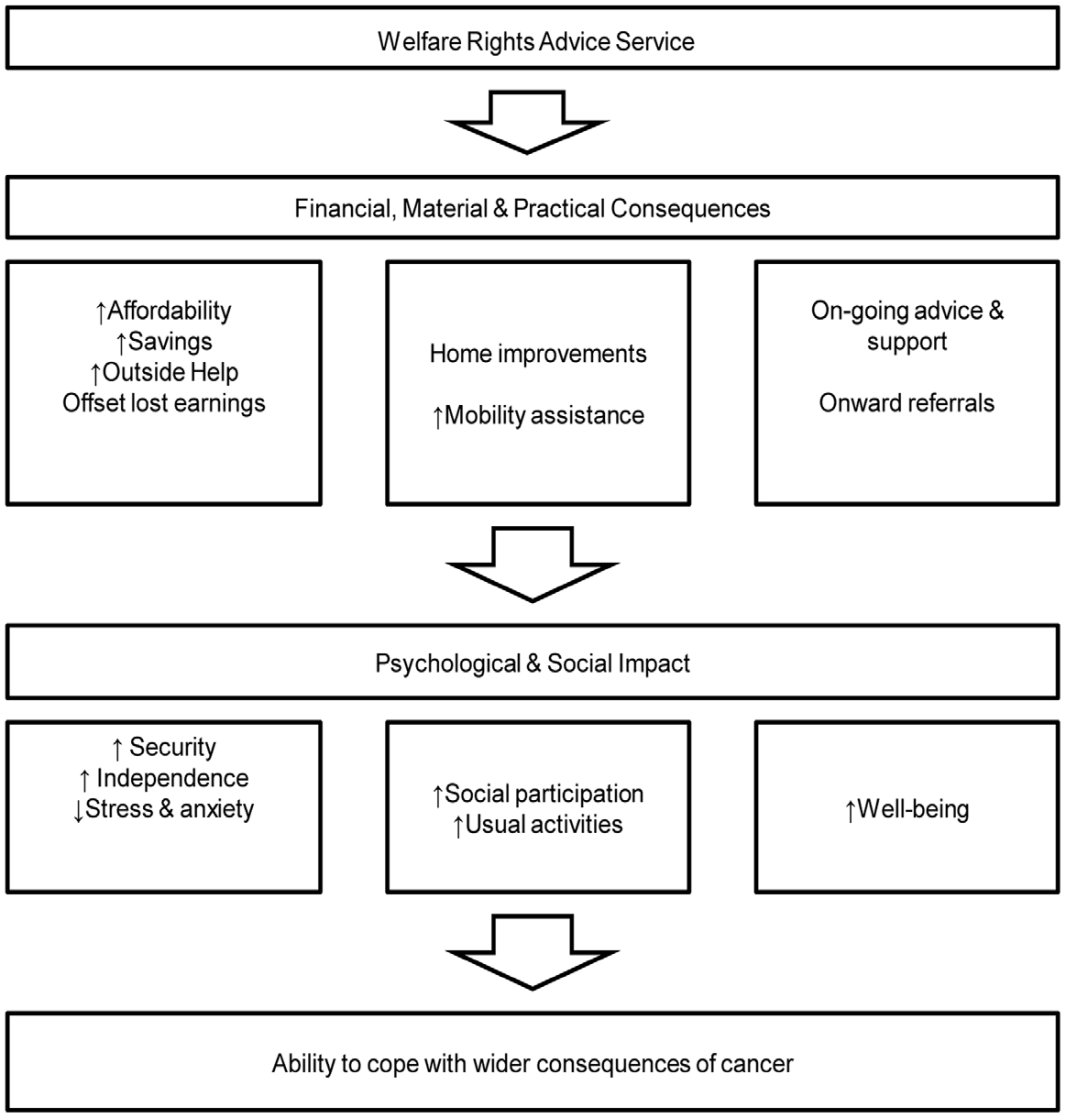
Perceived impact of welfare rights advice service for patients and carers affected by cancer, derived from qualitative study.

Drawing on these accounts, we devised a theoretical model (Figure 1) to explain how these immediate consequences impacted positively on wellbeing and assisted individuals to cope with the wider consequences of cancer. The immediate impact concerned direct financial and other material and practical consequences. These resulted in positive psychological and social impacts. Participants reported reduced levels of stress and anxiety related to financial difficulties.

I’ve now got no money worries, which has probably helped my health because I don’t have to worry about the bills … I just concentrate on getting better. (P078, male, aged 50–54).

Socially, the additional resources increased individuals’ capacity to engage in ‘normal’ or ‘routine’ activities such as going out for a drive, for a meal, taking children and grandchildren out or reciprocating for help received. Engaging in these routine activities was of great symbolic value during periods where the primary focus was on illness and treatment regimes. The cumulative effect was to remove or reduce anxieties about finance, enabling patients and carers to focus on dealing with cancer, which was beneficial physically, psychologically and socially and improved quality of life.

Without the extra [money], it would have been very, very hard to cope. I just know it would … I’m not saying it relieves your symptoms, but it keeps your head clear kind of thing, do you know what I mean? Because if your head’s all worried and jumbled with finances the rest of your body goes down doesn’t it? You know, because you stop eating for a start, you know you’re making yourself poorly. I do know it would have been desperate. (P017, female, aged 65–69 years).

#### Barriers to accessing the benefits system

Lack of knowledge about available benefits and eligibility criteria were the greatest barriers. Participants knew that there was a benefits system, but most had no idea what benefits existed, nor how to go about claiming them. Individuals already receiving benefits were no more likely to pursue a benefit claim independently than those not, nor were they more likely to be aware of their entitlement to benefit.

I wouldn’t have applied for them [benefits] off my own back because as I say, I didn’t know I was entitled to anything. (P036, female, aged 45–49 years).

General publicity about benefits in the form of leaflets or television advertising did not appear to alert participants to their entitlements.

I mean we see all these adverts on the TV about people aren’t claiming their benefits, but if you don’t know what the benefits is, it’s not very helpful. (Carer, female, retired).

Concern was expressed about the number of professionals who patients and carers were in contact with, but who did not alert them to benefit entitlements. A commonly held view was that, if no one had informed them, then they would not be entitled. Homeowners or those with savings often made assumptions that they would not be eligible for benefits.

Several participants had prior experience with the benefits system and described the experience as time consuming and complex. Some had received help and support to make claims in the past though many people described a lack of readily available information or, in some cases instances where they were given wrong information. Participants stated that when making enquiries about their eligibility for a particular benefit, they had not been alerted to other benefits that they may have been entitled to, particularly health related benefits.

Health status acted as a barrier since the impact of many cancer treatments was debilitating both physically and mentally and many participants were pre-occupied with cancer-related treatment, especially in the weeks following diagnosis. Participant accounts of fatigue, pain, nausea or inability to concentrate deterred individuals from the lengthy and complex process of establishing eligibility criteria and completing lengthy benefit applications without assistance.

Some participants expressed negative attitudes towards claiming benefits. Values of hard work, ‘making do’, pride and self-reliance emerged as attitudinal barriers to claiming. Many participants recounted how they had worked hard all their lives and avoided claiming benefits, despite periods of unemployment. Moreover, several related unpleasant dealings with the benefits system in the past. People with this mindset were reluctant to actively seek help, although when offered, did accept.

It’s just this stigma attached, oh you’re claiming this, but I think now I’ve got to the point where it does help, even with the shopping, so I would advise anyone, yes do claim if you need it, because it does help towards the financial side … you just have to swallow your pride … it’s really good they’ve got things in place like that. (P057, female, aged 35–39 years).

Compounding these views were beliefs about extent and severity of illness. Some felt that they were not ill enough and therefore not genuinely deserving of health-related benefits, despite in some cases serious illness and poor prognoses. These feelings were, for some, bound up with the process of accepting and dealing with illness; the receipt of benefits symbolising an inability to cope with the financial aspects of life that had previously been managed prior to becoming ill with cancer.

Well, it’s partly my attitude … I fight all along to try to assume that I can cope with [illness] easily … when I go along to the surgery or anywhere, hospital, whatever, I look around at all the other people in the waiting area and a I feel a bit of a fraud. (P015, male, aged 75–79 years, retired).

## Discussion

This is the first study assessing through qualitative interviews the extent to which routinely embedding assistance with financial and other aspects of social welfare impacts on cancer patients’ perceived quality of life and wellbeing. The service was widely accessible and referral could be from health care practitioners, local authority or voluntary sector practitioners or patients themselves. Following consultation with a welfare rights advisor and assessment of eligibility, 96 per cent of the claims made were successful resulting in significant additional resources for patients (median £70.30 ($109.74, €84.44) per week). Moreover, the service reached cancer sufferers living in areas of high socio-economic deprivation. The interviews demonstrate that accessing benefit entitlements had important financial, material and practical benefits, which in turn had important positive social and psychological consequences (Figure 1). Interviews revealed that many individuals did not know what assistance might be available to them or how to claim. Further barriers to accessing assistance unaided arose from the physical and psychological impact of the illness. The findings suggest that this intervention can offset some of the financial impact of a cancer diagnosis, assist people to cope with the wider consequences of cancer and positively impact on quality of life. Since people are now living for much longer periods with cancer, the effects of the illness on financial wellbeing are likely to be greater than has been the case hitherto [5]. Moreover, the stress resulting from the financial consequences of a cancer diagnosis and treatment is likely to be clinically important [4].

### Strengths and Limitations

To our knowledge, this is the first evaluation of a dedicated welfare rights advice service for cancer patients. The study population was diverse, including patients above and below national pension age with a wide range of cancer types. A major strength is the level of detail obtained about success rates and the range of financial and other benefits obtained for cancer patients and carers. As the sample was drawn from people accessing the welfare rights advice service, the findings are derived from people more likely to be affected by financial strain and stress after cancer, which does have implications for generalisability to the population with cancer as a whole. A further potential limitation is that those receiving additional resources may be overly positive about financial gains and therefore overestimate their impact. Nevertheless, financial strain associated with cancer is a common experience in the UK [11] and elsewhere [5] [41]. The sample included more women than men, probably due to a combination of higher levels of female poverty particularly in later life [42], and females being more likely to seek help than males [43]. We were unable to examine data on ethnicity, and cannot therefore draw any conclusions about UK ethnic minority groups or people whose first language was not English, although evidence suggests that these groups have even greater difficulties accessing benefits than the white population [44] and evidence from the US indicates that ethnic minority groups may have significantly greater need for financial assistance [45]. Evidence from health professionals is required to ascertain potential impact on clinical workload, but previous research indicates that health professionals are aware of the strain that financial difficulties can place on cancer patients and their households and appropriate services dealing with this aspect of the illness would be highly valued [46]. While the findings may be of less relevance to areas with less widespread and severe socio-economic deprivation, the study revealed that people from all socio-economic groups accessed the service, although more people from lower socio-economic groups both accessed and benefitted from the service. A further limitation is the extent to which this type of service might be applicable elsewhere, since welfare systems compensate differently for the financial impact of ill-health [47]–[48].

### Why Financial Consequences of a Cancer Diagnosis are Relevant to Health Care Practitioners

An earlier study into the psycho-social needs of cancer patients found that financial needs were most likely to be unmet and that for some of those experiencing financial hardship, ‘*this aspect of living with cancer was almost worse than the disease itself*’ [49] (p602). Loss of income, reduced savings, short or long-term unemployment, reduced occupational pension and additional costs associated with cancer treatment are the main financial consequences of a cancer diagnosis in many developed welfare states. In the USA, the extent to which individuals are covered by their insurance policies or eligible for state-funded health care is a key challenge [4] **.** This study suggests that at least some cancer patients struggle with the UK welfare system and the financial problems associated with their illness when left to deal with them unaided. Awareness of this aspect of cancer is important for two reasons. Firstly, timely advice needs to be given to those with a terminal diagnosis, ensuring that all possible assistance is in place without undue delay and that ongoing support can be offered to carers and other family members during the period of the illness and after death. Secondly, it is important that the increasing number of cancer survivors receive expert advice and guidance about negotiating welfare systems and, for those of working age, information and assistance about sickness benefits and returning to employment where possible [2] [46].

In the UK, it has been shown that delays in claiming welfare benefits result in a significant loss of income for terminally ill cancer patients [50] and lung cancer patients [20], despite regular contact with a range of health and social care professionals. Given that eligibility rules are more straightforward for people who are terminally ill, this suggests a failure to holistically address end of life issues.

### Implications for Health Care Practitioners

The findings of this study raise an important question concerning professional responsibilities; what is the duty of health care practitioners in relation to patients’ financial difficulties arising from ill-health? For clinical staff to offer such advice is neither appropriate nor practical. Within many health care systems it is often unclear who, if anyone, has responsibility or capacity for assisting patients to deal with the financial consequences of illness [4] [46] [1] [5]. Moreover, the boundaries between medical and social issues are blurred and there are often no routinely available services with the expertise to deal with economic, social and legal issues arising from ill-health [41]. A clear implication from this study is that expert advice and assistance to claim financial and other benefits needs to be *provided* by trained welfare rights advisors and not health care practitioners. But, the question remains who should bear responsibility for ensuring that this is addressed? In the UK, health care practitioners are obliged to undertake the ‘Holistic common assessment for supportive and palliative care needs for adults requiring end of life care’ [51] which has a domain on work and finance. A major improvement would be recognition that a single health care practitioner is responsible for co-ordinating referral onto appropriate services and the key role that finance may have for cancer patients. Recognising that financial stress is a likely consequence of cancer as well as onward referral to appropriate services, whether in the statutory or charitable sector, should become routine practice [4] [46]. Improved treatments and longer survival for cancer patients is reducing some of the differences between cancer and other chronic conditions. The findings of this study therefore have broader implications for services that could be routinely provided for other illnesses, given the financial costs associated with ill-health and disability [52]–[53].

This study has demonstrated the benefits to patients and carers when referral to welfare rights advice services becomes embedded within routine clinical practice. However, the findings coincide with a period of retrenchment in the European model of the welfare state [54]. Specifically in the UK, welfare reforms are likely to reduce the amount, type and duration of financial benefits that some individuals receive which will therefore adversely affect people with cancer [55]. Furthermore, public sector spending cuts will make it less, rather than more likely that appropriate welfare rights services will be available for patients [55].

### Conclusions

Our findings indicate that in addition to screening for financial problems, health professionals require access to good quality information, advice and advocacy services to which they can refer their cancer patients. Addressing the financial sequelae of a cancer diagnosis appears to have positive social and psychological consequences that could significantly enhance the clinical management of cancer and quality of life for cancer patients. Further research is needed to evaluate health outcomes definitely and assess cost-effectiveness.

## Supporting Information

Appendix S1 **Description of selected UK state welfare benefits claimed by the study population.**
(DOC)

## References

[1] Francoeur RB (2007) The influence of age on perceptions of anticipated financial inadequacy by palliative radiation outpatients. Patient Education and Counseling 69: 84–92. 17766078 10.1016/j.pec.2007.07.005 PMC2243225

[2] Wilson K, Amir Z (2008) Cancer and disability benefits: a synthesis of qualitative findings on advice and support. Psycho-Oncology 17: 421–429. 17828716 10.1002/pon.1265

[3] Pearce S, Kelly D, Stevens W (1999) ‘More than just money’ - widening the understanding of the costs involved in cancer care. Journal of Advanced Nursing 33: 317–379. 10.1046/j.1365-2648.2001.01673.x 11251724

[4] Francoeur RB (2001) Reformulating financial problems and interventions to improve psychosocial and functional outcomes in cancer patients and their families. Journal of Psychosocial Oncology 19: 1–20.

[5] Hanratty B, Holland P, Jacoby A, Whitehead M (2007) Review article: Financial stress and strain associated with terminal cancer - a review of the evidence. Palliative Medicine 21: 595. 17942498 10.1177/0269216307082476

[6] Wright EP, Kiely MA, Lynch P, Cull A, Selby PJ (2002) Social problems in oncology. British Journal of Cancer 87: 1099–1104. 12402148 10.1038/sj.bjc.6600642 PMC2376184

[7] Syse A, Tretli S, Kravdal O (2008) Cancer’s impact on employment and earnings - a population-based study from Norway. Journal of Cancer Survivorship 2: 149–158. 18792789 10.1007/s11764-008-0053-2

[8] Lauzier S, Maunsell E, Drolet M, Coyle D, Hebert-Croteau N, et al (2008) Wage losses in the year after breast cancer: Extent and determinants among Canadian women. J Natl Cancer Inst 100: 321–332. 18314472 10.1093/jnci/djn028

[9] de Boer AGEM, Taskila T, Ojajarvi A, van Dijk FJH, Verbeek JHAM (2009) Cancer survivors and unemployment: A meta-analysis and meta-regression. JAMA 301: 753–762. 19224752 10.1001/jama.2009.187

[10] Himmelstein DU, Warren E, Thorne E, Woolhandler S (2005) Illness and injury as contributors to bankruptcy. Health Affairs. doi: 10.1377/hlthaff.w5.63. 10.1377/hlthaff.w5.63 15689369

[11] Macmillan Cancer Relief (2004) The Unclaimed Millions. Available: www.macmillan.org.uk.showdocument.asp?id=443. Accessed 2008 Oct 1.

[12] Quinn MJ, Babb P, Brock A, Kirby L, Jones J (2001) Cancer trends in England and Wales, 1950–1999. Studies on Medical and Population Subjects No. 66. London: The Stationery Office.

[13] Shack L, Jordan C, Thomson CS, Mak V, Møller H, et al (2008) Variation in incidence of breast, lung and cervical cancer and malignant melanoma of skin by socioeconomic group in England. BMC Cancer 8: 271–281. 18822122 10.1186/1471-2407-8-271 PMC2577116

[14] Kogevinas M, Porta M (1997) Socio-economic differences in cancer survival: a review of the evidence. In: Kogevinas M, Pearce M, Susser M, Boffetta p, editors. Social Inequalities and Cancer: International Agency for Research on Cancer, WHO 177–206.

[15] Singh GK, Miller BA, Hankey BF, Edwards BK (2003) Area socioeconomic variations in the US, cancer incidence, mortality, stage, treatment and survival, 1975–1999. National Cancer Institute. 03–5417 03–5417.

[16] Ward E, Jemal A, Cokkinides V, Singh GK, Cardinez C, et al (2004) Cancer disparities by race/ethnicity and socioeconomic status. Cancer Journal for Clinicians 54: 78–93. 10.3322/canjclin.54.2.78 15061598

[17] Rosengren A, Wilhelmsen L (2004) Cancer incidence, mortality from cancer and survival in men of different occupational classes. European Journal of Epidemiology 19: 533–540. 15330125 10.1023/b:ejep.0000032370.56821.71

[18] The Marmot Review (2010) Fair Society, Healthy Lives. London: UCL. ISBN 978–0–9564870–0–1 1–242.

[19] National Audit Office (2005) Department of Work and Pensions: Dealing with the Complexity of the Benefits System. London: The Stationery Office.

[20] Chapple A, Ziebland S, McPherson A, Summerton N (2004) Lung cancer patients’ perceptions of access to financial benefits: A qualitative study. British Journal of General Practice 54: 589–594. 15296557 PMC1324838

[21] National Institute for Health and Clinical Excellence (2004) Improving Supportive and Palliative Care for People with Cancer (The Manual). London: National Institute for Clinical Excellence.

[22] Department of Health (2010) National Cancer Patient Experience Survey. Available: http://www.dh.gov.uk/en/publicationsandstatistics/publications/publicationsstatistics/DH_122516. Accessed 2011 Jan 18.

[23] Paris JAG, Player D (1993) Citizens advice in general practice. *BMJ* 306: 1518–1520. 8518682 10.1136/bmj.306.6891.1518 PMC1677949

[24] Acheson D (1998) Independent Inquiry into Inequalities in Health. London: HMSO.

[25] Fraser of Allander Institute (2003) The impact of welfare spending on the Glasgow economy. Glasgow: University of Strathclyde.

[26] Moffatt S, Scambler G (2008) Can welfare-rights advice targeted at older people reduce social exclusion? Ageing and Society 28: 875–899.

[27] Adams J, White M, Moffatt S, Howel D, Mackintosh J (2006) A systematic review of the health, social and financial impacts of welfare rights advice delivered in health care settings. BMC Public Health 6: 81. 16571122 10.1186/1471-2458-6-81 PMC1440855

[28] Association of Public Health Observatories and Department of Health (2009) Health summary for County Durham UA. Available: http://www.apho.org.uk/default.aspx?QN=HP_METADATA&AreaID=71130. Accessed 2010 Jan 21.

[29] Northern and Yorkshire Cancer Registry and Information Service (2008) County Durham PCT Factsheet. Available: http://www.nycris.nhs.uk/uploads/doc576_109_PCT%20Factsheet%20NECN%20-%2010%205ND%20County%20Durham%20PCT.pdf. Accessed 2010 Jan 21.

[30] Indices of deprivation (2007) Available: http://www.communities.gov.uk/communities/neighbourhoodrenewal/deprivation/deprivation07/.Accessed 2009 May 6.

[31] Statistical Package for Social Science (2009) V17.0 for Windows 17.0 ed. Chicago, Illinois.

[32] Green J, Thorogood N (2009) Qualitative Methods for Health Researchers. London: Sage.

[33] Coyne IT (1997) Sampling in qualitative research. Purposeful and theoretical sampling: merging or clear boundaries? J Advanced Nursing 26: 623–630. 10.1046/j.1365-2648.1997.t01-25-00999.x 9378886

[34] Denzin NK (1978) The Research Act. New York: McGraw-Hill Book Company.

[35] QSR International (2007) NVivo 7: Computer Assisted Software for Qualitative Analysis. Available: http://www.qsrinternational.com/support_resource-articles_detail.aspx?view=88. Accessed 2012 Jul 18.

[36] Ritchie J, Lewis J, editors (2003) Qualitative Research Practice. A Guide for Social Scientists. London: Sage.

[37] Glaser B, Strauss A (1967) The Discovery of Grounded Theory. Chicago: Aldine.

[38] Silverman D (2000) Doing qualitative research. London: Sage.

[39] Clayman SE, Maynard DW (1995) Ethnomethodology and Conversation Analysis. In: ten Have P, Psathas G, editors. Situated Order: Studies in the Social Organisation of Talk and Embodied Activities. Washington, D.C.: University Press of America. 1–30.

[40] Barbour RS (2001) Checklists for improving rigour in qualitative research: the case of the tail wagging the dog? British Medical Journal 322: 1115–1117. 11337448 10.1136/bmj.322.7294.1115 PMC1120242

[41] Cook K, Dranove D, Sfekas A (2010) Does major illness cause financial catastrophe? Health Services Research 45: 418–436. 19840132 10.1111/j.1475-6773.2009.01049.x PMC2838153

[42] Arber S, Ginn J, editors (1995) Connecting gender and ageing. Buckingham: OUP.

[43] Men’s Health Forum (2009) Challenges and Choices. Improving health services to save men’s lives. Available: http://www.menshealthforum.org.uk/content/nmhw-2009-policy-webpdf. Accessed 2010 Apr 4.

[44] Moffatt S, Mackintosh J (2009) Older people’s experience of proactive welfare rights advice: qualitative study of a South Asian community. Ethnicity and Health 14: 5–25. 19152156 10.1080/13557850802056455

[45] Welch LC, Teno JM, Mor V (2005) End of life care in black and white: race matters for medical care of dying patients and their families. Journal of the American Geriatric Society 53: 1145–1153. 10.1111/j.1532-5415.2005.53357.x 16108932

[46] Mayer DK, Reiner A (2009) The costs of cancer. Clinical Journal of Oncology Nursing 13: 255–256. 19502181 10.1188/09.CJON.255-256

[47] Eikemo TA, Bambra C (2008) The welfare state: a glossary for public health. Journal of Epidemiology and Community Health 62: 3–6. 18079325 10.1136/jech.2007.066787

[48] Brennenstuhl S, Quesnel-Vallee A, McDonough P (2011) Welfare regimes, population health and health inequalities: a research synthesis. JECH Online First: 1–13. 10.1136/jech-2011-200277 22080814

[49] Soothill K, Morris SM, Harman J, Francis B, Thomas C, et al (2001) The significant unmet needs of cancer patients: probing psychosocial concerns. Support Care Cancer 9: 597–605. 11762970 10.1007/s005200100278

[50] Nosowska G (2004) A delay they can ill afford: delays in obtaining Attendance Allowance for older, terminally ill cancer patients, and the role of health and social care professionals in reducing them. Health & Social Care in the Community 12: 283–287. 15272883 10.1111/j.1365-2524.2004.00496.x

[51] National Cancer Action Team (2007) Holistic common assessment of supportive and palliative care needs for adults with cancer. King’s College London.

[52] Argyle E (2001) Poverty, disability and the role of older carers. Disability and Society 16: 585–595.

[53] Smith R, Sim J, Scharf T, Philipson C (2004) Determinants of quality of life amongst older people in deprived neighbourhoods. Ageing & Society 24: 793–814.

[54] McKee M, Stuckler M (2011) The assualt on universalism: how to destroy the welfare state. BMJ 343. 10.1136/bmj.d7973 22187190

[55] Wintour P (2011) Welfare bill “will penalise cancer patients”. The Guardian, 1.

