# Supplementary material for: Addressing the Financial Consequences of Cancer: Qualitative Evaluation of a Welfare Rights Advice Service
Source: PLoS One. 2012 Aug 10;7(8):e42979. doi: 10.1371/journal.pone.0042979 (PMC3416776; doi:10.1371/journal.pone.0042979)
Supplement: Appendix S1 — Description of selected UK state welfare benefits claimed by the study population. (DOC) [file pone.0042979.s001.doc]

**Appendix S1**

Description of selected UK state welfare benefits claimed by the study population

| **Attendance Allowance** | Non-means tested benefit paid to claimants over 65 who are physically or mentally disabled and meet specific health related criteria. There is a lower and higher rate of the benefit. |
| --- | --- |
| **Carers Allowance** | A non-means tested benefit to help people who look after someone who is disabled. Certain exclusions apply, for people in employment or education. |
| **Council Tax Benefit** | A benefit paid to people whose income and capital (savings and investments) are below a certain level. |
| **Disability Living Allowance** | Non-means tested benefit paid to claimants under 65 who have a physical disability or a mental disability (including learning disabilities) or both. The care component has three rates, while the mobility component has two rates. |
| **Employment Support Allowance** | This is paid to those people who have a limited capability to work due to disability or ill health. There are two elements: contributory which is linked to national insurance contributions and income related which is means tested. Claimants are placed in either the support group or the work related activity group, which determines the amount received. |
| **Macmillan Grants** | This grant is a one-off lump sum payment for adults, young people or children with cancer, to cover a wide range of practical needs. This can include things such as heating bills and extra clothing. It is generally for people on low incomes or with reduced personal savings. |
| **Pension Credit**  **(Guarantee)** | This benefit is paid to people who have reached the minimum qualifying pension age and guarantees a minimum income. |
